# Calcium Silicate/Chitosan-Coated Electrospun Poly (Lactic Acid) Fibers for Bone Tissue Engineering

**DOI:** 10.3390/ma10050501

**Published:** 2017-05-05

**Authors:** Chu-Jung Su, Ming-Gene Tu, Li-Ju Wei, Tuan-Ti Hsu, Chia-Tze Kao, Tsui-Han Chen, Tsui-Hsien Huang

**Affiliations:** 1 Antai Medical Care Cooperation, Antai Tian-Sheng Memorial Hospital, Pingtung City 928, Taiwan; a085085@mail.tsmh.org.tw; 2 School of Dentistry, China Medical University, Taichung City 404, Taiwan; mgtu@mail.cmu.edu.tw; 3 3D Printing Medical Research Center, China Medical University Hospital, China Medical University, Taichung City 404, Taiwan; lijuwei928@gmail.com (L.-J.W.); nakohsu@gmail.com (T.-T.H.); 4 School of Dentistry, Chung Shan Medical University, Taichung City 404, Taiwan; ctk@csmu.edu.tw; 5 Department of Stomatology, Chung Shan Medical University Hospital, Taichung City 404, Taiwan; 6 Institute of Oral Science, Chung Shan Medical University, Taichung City 404, Taiwan; n8902610@gmail.com

**Keywords:** poly (lactic acid), chitosan, calcium silicate, tissue engineering, osteogenesis

## Abstract

Electrospinning technology allows fabrication of nano- or microfibrous fibers with inorganic and organic matrix and it is widely applied in bone tissue engineering as it allows precise control over the shapes and structures of the fibers. Natural bone has an ordered composition of organic fibers with dispersion of inorganic apatite among them. In this study, poly (lactic acid) (PLA) mats were fabricated with electrospinning and coated with chitosan (CH)/calcium silicate (CS) mixer. The microstructure, chemical component, and contact angle of CS/CH-PLA composites were analyzed by scanning electron microscopy, X-ray diffraction, and Fourier transform infrared spectroscopy. In vitro, various CS/CH-coated PLA mats increased the formation of hydroxyapatite on the specimens’ surface when soaked in cell cultured medium. During culture, several biological characteristics of the human mesenchymal stem cells (hMSCs) cultured on CS/CH-PLA groups were promoted as compared to those on pure PLA mat. Increased secretion levels of Collagen I and fibronectin were observed in calcium silicate-powder content. Furthermore, with comparison to PLA mats without CS/CH, CS10 and CS15 mats markedly enhanced the proliferation of hMSCs and their osteogenesis properties, which was characterized by osteogenic-related gene expression. These results clearly demonstrated that the biodegradable and electroactive CS/CH-PLA composite mats are an ideal and suitable candidate for bone tissue engineering.

## 1. Introduction

Tissue engineering is an emerging trend that has been extensively studied in past few decades. It aims to develop biological materials that is able to restore, support, and promote tissue and organ regeneration, which involves cell recruitment, growth, proliferation, and differentiation [1]. Electrospinning technology allows for the fabrication of nano- or microfibrous fibers with inorganic and organic matrix and is widely applied in bone tissue engineering, as the process allows for precise control over the shapes and structures of the fibers [2,3]. Also, the simplicity and ease of functionalization of this technique makes it even more attractive. Recent reviews focus on the types of materials that can be eletrospun, and there is an increasing interest in the use of synthetic biodegradable polymers [4] that were approved by the US Food and Drug Administration, such as poly glycolide acid (PGA), poly caprolactone (PCL) [5], poly lactic acid (PLA) [2,6], and PLGA [7]. However, the list is non-exhaustive, and other suitable candidates for electrospinning include several natural polymers such as collagen, chitosan, silk, and alginate [8,9]. Among all the biodegradable materials, PLA is one of the most promising and common bio-polymers [2,6] that can be used in textile, drug-carrier, and implant applications. Recent studies indicate that PLA-based biomaterials have been used in the automotive, communication, and electronic industries, where a higher level of durability is required. Despite its excellent physical properties, PLA is a strongly hydrophobic material that exhibits weak cell-cell interactions and cell signaling, which thus greatly reducing its suitability as a biomaterial [10].

Natural bone has an ordered composition of organic fibers with a dispersion of inorganic apatite among them [11]. The compositions of natural bone serve as an inspiration to design a novel organic-inorganic hybrid bone substitute [12]. Chitosan (CH) is a positively charged natural polymer that is obtained by partial deacetylation (with enzymatic or alkaline treatment) of chitin [13]. Chitosan is a suitable biomaterial for various clinical applications, and the benefits of chitosan include high biocompatibility, low inflammatory responses from host, antibacterial properties, and high biodegradability [13]. CH can be used as a temporary scaffolding support for tissue growth and regeneration [14,15]. There is a high level of resemblance between the component and chemical structure of CH and the extracellular matrix of bones and cartilages [14]. In addition, CH-based scaffolds also demonstrated good osteoconductivity, which aids in promoting both in vitro and vivo hard tissue regeneration [16]. Despite its excellent biological properties, CH exhibits low mechanical properties, limiting its suitability for orthopedic implants [17]. Therefore, a proposed novel approach for a CH/ceramics scaffold is a better candidate for bone tissue engineering due to its excellent physicochemical and biological properties [18,19].

Calcium silicate (CS)–based ceramics have received great attention and are commonly used in hard tissue engineering due to a higher level of biocompatibility, bioactivity, and biodegradability [20,21,22]. CS-based materials displayed slower degradation and anti-bacterial behavior, especially in the early stages, making it a suitable candidate for clinical endodontic use [23]. CS is hydrophilic, thus enabling it to promote hydroxyapatite movement to the outer layers, which aids in promoting cell adhesion, proliferation, and differentiation. This biological property makes CS a promising biomaterial for bone tissue regeneration [24]. Previous studies show that calcium silicate–based materials can enhance osteogenesis differentiation from primary cells such as human mesenchymal stem cells (hMSCs), human periodontal ligament cells (hPDLs), and human dental pulp cells (hDPCs) [22,25,26,27]. It has been further reported that extracellular Ca ion has a significant effect on osteoblast proliferation and differentiation. In addition, Si is reported to be an essential factor in the early stages of hard tissue regeneration and calcification such as bone and cementum [28,29].

The aim of this study was to create a novel bone substitute composed of CS/CH-coated PLA materials. The physicochemical properties of four types of CS/CH-coated PLA electrospun substrate were characterized. A one-step process was done by mixing CS powder directly onto CH coatings. CS/CH-coated specimens were analyzed with X-ray powder diffraction (XRD) and Fourier transform infrared spectroscopy (FTIR). In addition, the rates of ECM protein adsorption and short-term human mesenchymal stem cells (hMSCs) adhesion were analyzed. Last, the proliferation and osteogenic differentiation of hMSCs were analyzed to measure the efficacy of the CS/CH coatings. 

## 2. Materials and Methods

### 2.1. Fabrication of PLA Nanofiber Mat

PLA nanofiber was fabricated via a modified electrospinning process [30]. In short, PLA (Mn = 146,000, Nature Works LLC, Minnetonka, MN, USA) was dissolved in a chloroform/DMSO mixture (75:25, *v*/*v*) at room temperature for 24 h. The electrospinning machine consists of a high voltage power supply, micro-pump syringe, and an aluminum collection board. The 6% PLA working solution was directly electrospun onto the aluminum foil-covered collector. A 5 mL syringe fitted with 23 G injection needle was used to fill the PLA solution, and the injection rate was 0.5 mL/h. The distance between the needle and the collector was 18 cm. An electrospinning voltage was applied to the needle using a high-voltage power supply at 21 kV.

### 2.2. Calcium Silicate/Chitosan Coating

Appropriate amounts of CaO (Sigma-Aldrich, St. Louis, MO, USA), SiO_2_ (High Pure Chemicals, Saitama, Japan), and 5% Al_2_O_3_ (Sigma-Aldrich) green materials were mixed and sintered at 1400 °C for 2 h by a high-temperature furnace. To this was added 99.5% ethyl alcohol, and the mixture was ground using a centrifugal ball mill (S 100, Retsch, Hann, Germany) for 6 h.

The acidulated CS powder was then prepared. In brief, 10 g of CS powder were stirred in 100 mL of 0.1 N acetic acid for 12 h and was then washed by deionized water thrice. Then, the CS suspensions were filtered through 0.22 μm filter paper and dried in an oven at 60 °C. 0.25 g of chitosan powder was dissolved in 100 mL of 0.1 N acetic acid at room temperature for 24 h under magnetic stirring. The acidulated CS powder was then dissolved in chitosan solution for a day. The CS solution concentration used in this study were 0%, 0.05%, 0.10%, and 0.15% (referred to as CS0, CS5, CS10, and CS15, respectively). 500 μL of CS/chitosan solution were covered on PLA mat for 3 h and was then rinsed with distilled water thrice and dried in an oven at 40 °C.

### 2.3. Characterization of CS/CH-PLA Mat

Distilled water was added to the CS/CH-coated PLA mats drop by drop and the pictures of contact angles were taken using a digital camera after an elapsed time of 30 s. An autopipette with meter was used to ensure that the volume of the deionized water droplets was the same (5 μL) for each sample. The results were evaluated using ImageJ software (National Institutes of Health) to analyze the water contact angle. Phase composition of CS/CH-coated PLA mat was measured using XRD (Bruker D8 SSS, Karlsruhe, Germany). The operation was conducted under 30 kV and 30 mA at a scanning speed of 1°/min. The chemical structure was evaluated using FTIR (Bruker Vertex 80v, Ettlingen, Germany) to observe the transformation of the chemical groups, and the spectra were collected at a range of 800 and 2000 cm^−1^ with a high resolution of 1 cm^−1^. The microstructure of the pure PLA mats and CS/CH-coated PLA composites were observed using a scanning electron microscope (SEM; JSM-6700F, JEOL, Nishiwaki, Japan).

### 2.4. Weight Loss and Ion Concentration

Degradation rate is considered as the important factor for evaluation of bone graft and it was analyzed by monitoring for the weight loss of these samples by soaking in Dulbecco’s modified Eagle’s medium (DMEM, Caisson, North Logan, UT, USA) at 37 °C. These CS/CH-coated PLA mats were dried at 60 °C and a balance was used to measure their weight before and after the soaking. Each group had six samples at every time point. The Ca, Si, Mg, and P ions concentration released from composites on SBF were analyzed using an inductively coupled plasma-atomic emission spectrometer (ICP-AES; Perkin-Elmer OPT 1MA 3000DV, Shelton, CT, USA).

### 2.5. Cell Adhesion and Proliferation 

The CS/CH-PLA samples were sterilized and soaked in 75% ethanol under ultraviolet light for 30 min before conducting cell experiments. The hMSCs were obtained from Sciencell Research Laboratories (Sciencell, Carlsbad, CA, USA) and commercial cultured medium (Sciencell) at passage 4–7 was used for cell culture. The hMSCs were directly cultured on the sterilized specimens (104 cells/well) in a 24-well plate and were incubated at 37 °C in a 5% CO_2_ atmosphere for short (adhesion) and long (proliferation) time. At the end of the culture time-point, the cultured medium was discarded and the specimens were washed twice with cold phosphate buffered saline (PBS, Caisson). Samples were covered with approximately 400 µL of DMEM without FBS and PrestoBlue^®^ (Invitrogen, Grand Island, NY, USA) solution at a ratio of 1:9. Samples were incubated at 37 °C for 1 h before analysis. The solution (100 µL) in each well was then transferred to a new 96-well plate and analyzed using a microplate reader (Tecan Infinite 200^®^ PRO) at 570 nm with a reference wavelength of 600 nm. hMSCs cultured on tissue culture plates without samples were used as control (Ctl) group. All data was collected in triplicate from separate experiments and displayed in terms of optical density.

### 2.6. Protein Adsorption on Substrates 

After being cultured for different periods of time, the amount of collagen (COL) and fibronectin (FN) secreted from cells to the specimens’ surface were analyzed using ELISA assay. Trypsin-EDTA solution (Cassion) was used to detach the cells from the surface after being washed with cold PBS thrice. Samples were then washed for three times with PBS-T (PBS containing 0.1% TWEEN-20), followed by the addition of 5% bovine serum albumin (BSA; Gibco, Carlsbad, CA, USA) in PBS-T for 1 h to stop the activity of trypsin. Primary antibodies were diluted at a ratio of 1:500 followed by incubating the mats with anti-human β-actin, anti-human COL I or anti-FN antibody (GeneTex, San Antonio, TX, USA) for 2 h at room temperature under shaking condition (25 rpm). Afterward, specimens were washed thrice with PBS-T for 5 min and incubated with horseradish peroxidase (HRP)-conjugated secondary antibodies for 1 h at room temperature under shaking condition. The samples were then washed thrice with PBS-T for 10 min respectively. One-Step Ultra TMB substrate (Invitrogen, Carlsbad, CA USA) was added to the wells and developed for 30 min at room temperature in the dark, after which an equal volume of 2 M H_2_SO_4_ was added to stop and stabilize the oxidation reaction. The colored products were then transferred to new 96-well plates and were read using a multiwell spectrophotometer at 450 nm with reference at 620 nm, according to the manufacturer’s recommendations. All experiments were carried out in triplicate. Additionally, β-actin antibodies were used as control group.

### 2.7. Cell Morphology

One day and 3 h after the hMSCs were cultured on CS/CH-PLA mats, the composites were washed three times with cold PBS and fixed in 2% glutaraldehyde (Sigma, St. Louis, MO, USA) for 2 h at room temperature, after which they were dehydrated using a graded ethanol series for 15 min at each concentration and dried with liquid CO_2_ using a critical point dryer device. The dried samples were mounted on the holder, coated with gold, and viewed using SEM. In addition, the hMSCs were cultured on composites for immunofluorescent staining at days 3 and 7. Cells and specimens were soaked in 4% paraformaldehyde for 30 min and permeabilized using 0.1% Triton X-100 for 20 min at room temperature. Then, the samples were treated with 1.5% bovine serum albumin for 1 h. Alexa Fluor-488-conjugated phalloidin (green color) was added to the cells and they were then incubated and shook for 1 h at room temperature. These specimens were then washed with TBS-T thrice and photographed under indirect immunofluorescence using a Zeiss Axioskop 2 microscope (Carl Zeiss, Thornwood, NY, USA).

### 2.8. Real-Time PCR

This study measured the expressions of osteogenic-related gene, such as collagen I (COL), alkaline phosphatase (ALP), osteopontin (OPN), and osteocalcin (OC) of hMSCs (104 cells/well) cultured on CS/CH-PLA composites at day 3 and day 7. All the RNA of hMSCs was extracted using TRIzol reagent (Invitrogen) and was analyzed using real time-polymerase chain reaction (RT-PCR) method. In short, all the RNA (400 ng) in each specimen was used for the synthesis of complementary DNA using cDNA Synthesis Kit (GenedireX, Las Vegas, NV, USA), conducted with reference to the manufacturer’s instructions. RT-PCR primers were designed based on cDNA sequences from the NCBI sequence database. Then, the SYBR Green qPCR Master Mix (Invitrogen) was used for detection and the target mRNA expressions were assayed on the ABI Step One Plus real-time PCR system (Applied Biosystems, Foster City, CA, USA). 

### 2.9. Statistical Analysis

A one-way analysis of variance (ANOVA) was used to consider the significance of the differences between the means in the measured data. Comparisons of the sample means were performed using Scheffe’s multiple comparison test. In all cases, the results were considered statistically significant if the *p*-value was < 0.05.

## 3. Results and Discussion

### 3.1. Characterization of CS/CH-Coated PLA Mats

**Figure 1:**
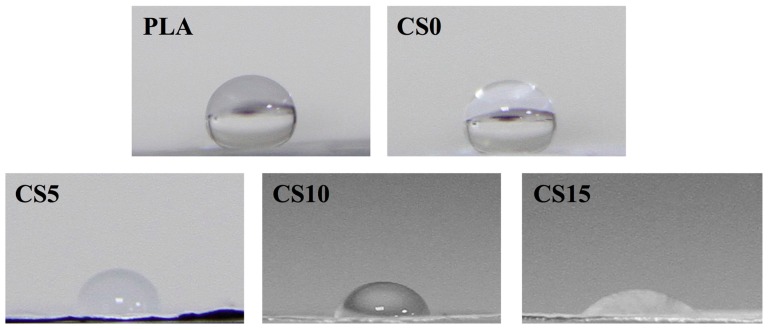
Water contact angle of different various calcium silicate/chitosan-coated PLA mats.

The hydrophilic behavior of pure PLA and CS/CH-coated PLA mats were tested by measuring the contact angle as shown in Figure 1. High contact angle (>100°) of pure PLA and HA-coated (CS0) mat specimens were observed by comparing with CH/HA-coated PLA specimens. There is no significant effect on the hydrophilicity of the PLA mats with CH coating. The contact angle of prepared CS/CH-coated PLA mat specimens was decreased from 91.2 ± 3.8°, 79.3 ± 3.2° to 35.1 ± 2.1° with every increasing coating concentration of 0.05% CS from 0.05% CS (CS5), 0.1% CS (CS10) to 0.15% CS (CS15), respectively. Concentration of CS coating and hydrophilic properties were correlated exponentially. The data indicated that the CS0 specimen is hydrophobic while the CS/CH coated mat with matrix precipitate are extremely hydrophilic, which also further indicate that cell adhesion and growth can be affected if cells are to be cultured on specimens within this range of contact angle [31].

**Figure 2:**
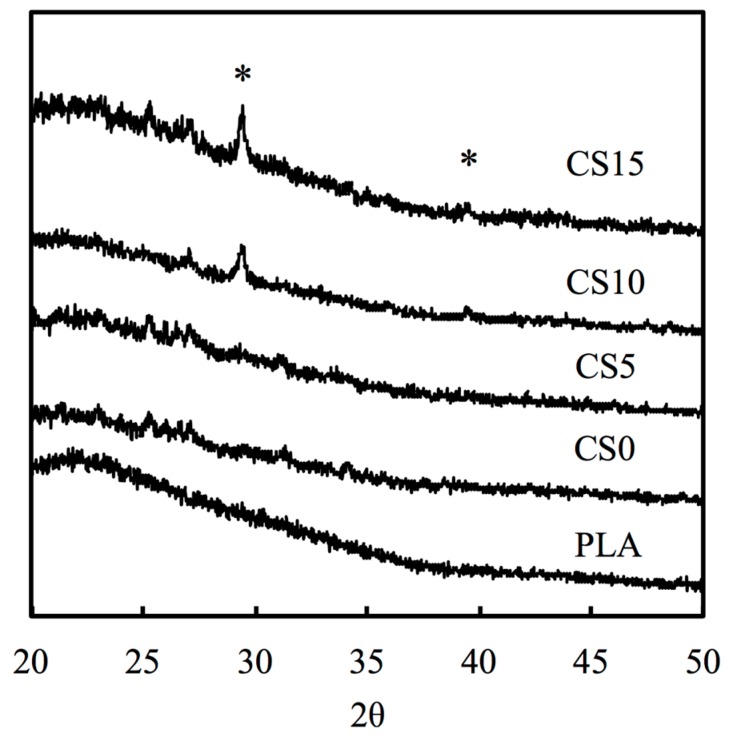
X-ray powder diffraction patterns of CS/CH-coated PLA mats. The marker indicates the calcium silicate.

**Figure 3:**
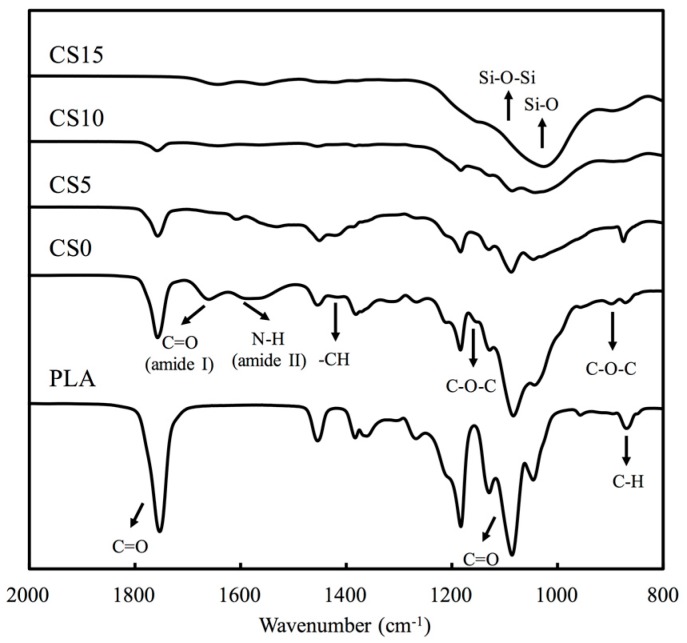
Fourier transform infrared spectra survey of various specimens.

Figure 2 shows the X-ray powder diffraction (XRD) patterns of the chitosan (CH)/calcium silicate (CS)-coated poly (lactic acid) (PLA) mat. There were no CS-related peaks noted in pure PLA and CS0. Peaks can be observed in CS-contained specimens and there were identical peaks observed in CS10 and CS15. The main peaks were approximately 2θ = 29.4° and 2θ = 39.2°, which were characteristic peaks of CS [32]. As seen on Figure 2, CS concentration on the surface of PLA mat were directly proportionate to the peak intensity of CS. Figure 3 shows the characteristic peaks of PLA and CS/CH-coated PLA mats from FTIR spectra. The spectra of PLA showed intense absorbance at 1750 cm^−1^, 1100 cm^−1^ due to the C=O and C–O stretch. The presence of chitosan was substantiated by the bands in the 1585 cm^−1^ and 1655 cm^−1^ regions due to the presence of NH2 scissoring and C=O stretching peaks, respectively. The broad peaks at 1010 cm^−1^ and 1051 cm^−1^ were due to the C–O stretching vibration in chitosan. Coating of increasing CS concentration makes the two peaks less evident. The broadest peak in specimen CS15 from 1000 to 1350 cm^−1^ corresponded to the Si–O and Si–O–Si stretch which is a cause of increasing content.

### 3.2. Bioactivity

**Figure 4:**
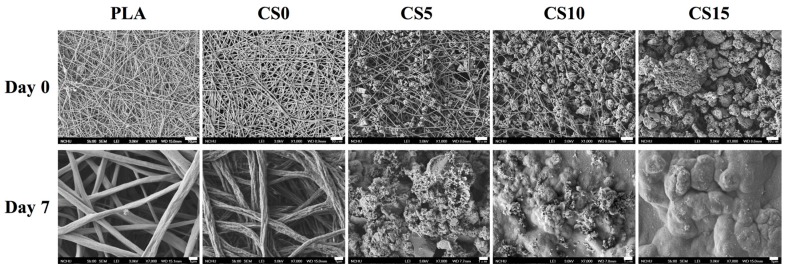
Surface scanning electron microscopy (SEM) images of the specimens before and after immersion in Dulbecco’s modified Eagle’s medium (DMEM).

Figure 4 shows the microstructure of the PLA and CS/CH-coated mat samples by SEM. After seven days of immersion in DMEM, there were no apatite precipitation observed on the PLA and CS0 samples. The amount of induced hydroxyapatite precipitation on the composites surface were increased with increasing CS concentration in the CS/CH matrix. As for the CS15 specimen, there were large amount of apatite observed which can be seen as a thick outer layer on the PLA mats. In previous study, the Si–OH functional groups around CS-based substrate were proven to act as nucleation centers for hydroxyapatite precipitation [24]. The amount of apatite precipitated on the in DMEM had been verified to be helpful in estimating the binding force between the materials and bone in vitro. 

**Figure 5:**
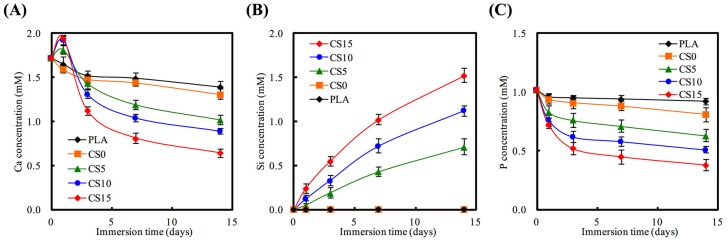
(**A**) Ca; (**B**) Si; and (**C**) P ions concentration of DMEM after specimens soaking for different times.

The concentrations of Ca, Si, and P ions released in various immersion time periods are shown in Figure 5. CS/CH coated PLA mats had an effect on the release rates of Ca ion. The Ca ion release rates increased with increasing CS coating content after the first day of immersion, followed by a gradual decrease of concentration to below 1.0 mM. PLA specimens without CS coating displayed a slower release rate as compared to the rest. However, as shown in Figure 5A, the higher the initial concentration of CS coating, the lower the final Ca concentration. On the other hand, the higher the initial concentration of CS coating, the samples displayed a lower final P concentration and a higher Si concentration. Figure 5B shows the concentration and rate of Si ion release. Si ion was continuously released for 14 days at a steady rate. After being immersed in DMEM for 14 days, Si ion concentration in the medium of CS0, CS5, CS10, and CS15 were 0, 0.71, 1.12, and 1.52 mM, respectively. Several studies showed that the CS-contained materials not only exhibit excellent bioactivity [23], but it also promotes odontogenesis and angiogenesis of primary cells [33,34]. In contrast, P ion significantly declined in all groups with CS/CH matrix at all immersion time-points (Figure 5C). These results demonstrated that CS15, with an optimum bioactivity, was expected to form a chemical bond between CS/CH-PLA mats and hard tissue through the thick hydroxyapatite layer.

**Figure 6:**
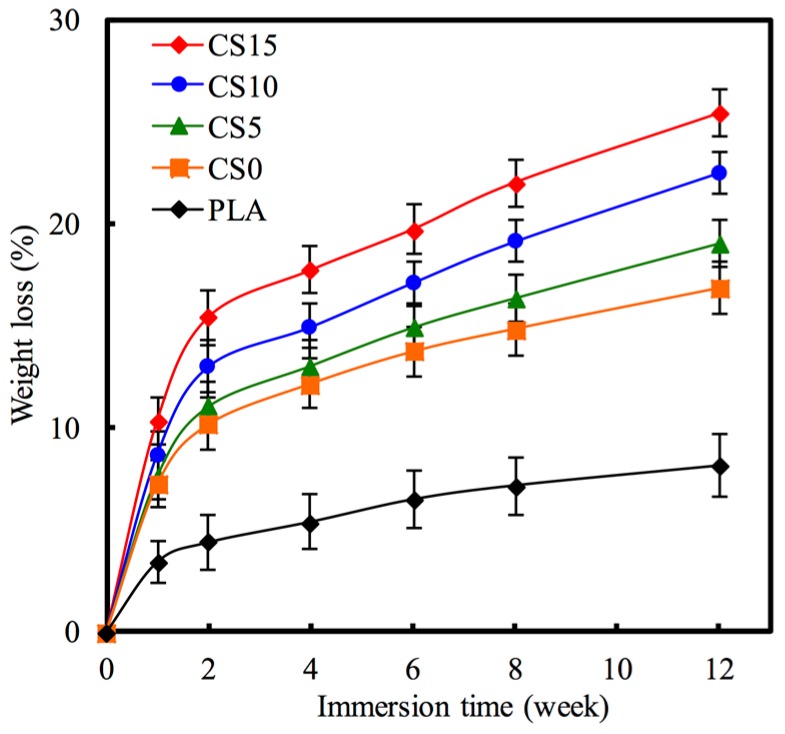
The weight loss of CS/CH-coated PLA mats immersed in DMEM for different time-points.

Biodegradation rate of a biomaterial can be justified by its weight loss through dissolubility in DMEM solution. Figure 6 shows the weight loss of each specimen after immersion in DMEM solution from one to 12 weeks. All specimens displayed a high rate weight loss during the first two weeks of soaking, after which the rate gradually slowed down. After soaking for four weeks, the pure PLA specimen showed lowest degradation rate compared to the others with a total weight loss of 5.38%. The CH and CH/CS coated PLA mat specimens showed higher degradation rate as compared to PLA mat specimens. CS15 showed the highest degradation rate among all specimens and the weight loss reached 22.01% after 8 weeks. Significant differences (*p* < 0.05) in weight losses of 8.12%, 16.82%, 19.01%, 22.51%, and 25.41% were observed for PLA, CS0, CS5, CS10, and CS15 mats at the final time-point respectively. This implies that these biomedical materials with different degradation times can be used for different clinical uses [24].

### 3.3. Protein Adsorption

**Figure 7:**
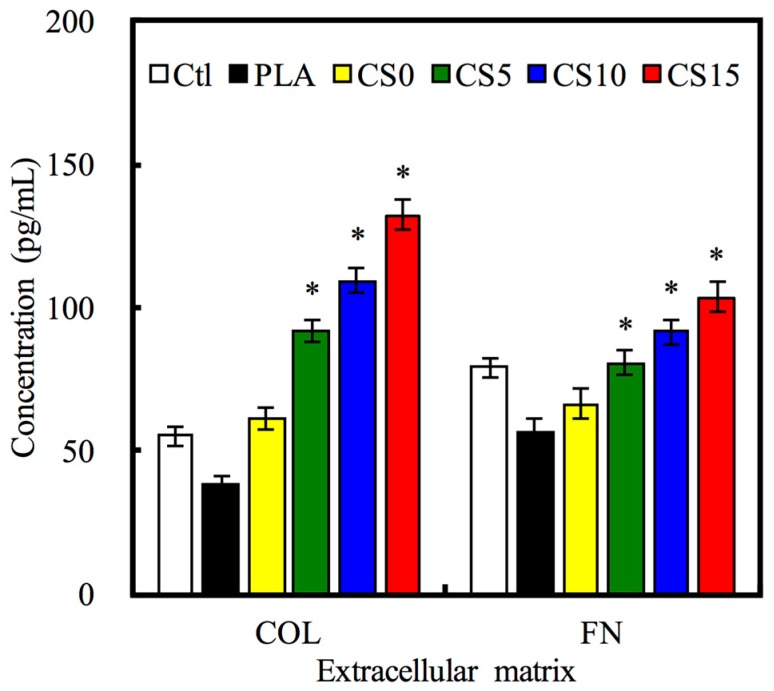
Collagen (COL) I and fibronectin (FN) adsorbed on CS/CH-coated PLA mats surface by as human mesenchymal stem cells (hMSCs) secretion for 3 h. “*” indicates a significant difference (*p* < 0.05) compared to CS0.

The effect of CS/CH-coated PLA on the absorption of Col I and FN secreted from hMSCs was also considered. At 3 h after cell seeding, the amount of Col I adsorption was significantly higher (*p* < 0.05) on the highest CS-contained (CS15) mat than on the PLA mat (Figure 7). Comparing with C0, Col I absorption increased from 51%, 79% to 117% for CS5, CS10, and CS15, respectively. In CS-contained groups, there was a decrease in pH of the micro-environment thus causing the collagen to assemble on the specimen surface [34]. The covalent immobilization of ECM protein on the material was shown by the uniformity and stability of the protein on the specimens’ surface [35]. The Si–OH groups and silicate not only supplied active sites for nucleation of hydroxyapatite, but also contributed to their cell behavior, such as adhesion and proliferation [36]. COL I is the most abundant protein found in nature bone, characterized by a triple helix structure with repetitive amino acids that allows interaction with various biomolecules that regulate cell adhesion and cell function [37,38]. The various ECM proteins on the substrate which regulate these cell adhesion-related proteins were activated and induced signaling pathways required for specific cell behaviors [39]. 

### 3.4. Cell Adhesion and Proliferation

**Figure 8:**
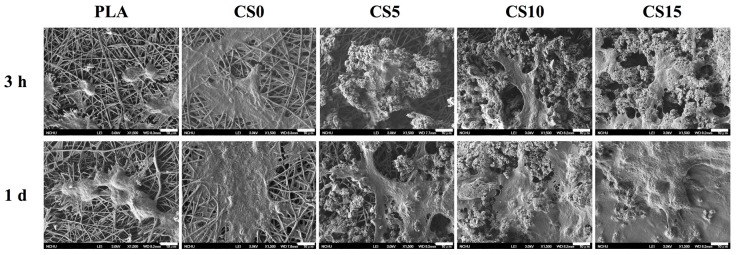
SEM images of hMSCs adhered on CS/CH-coated PLA mats for 3 h and one day.

**Figure 9:**
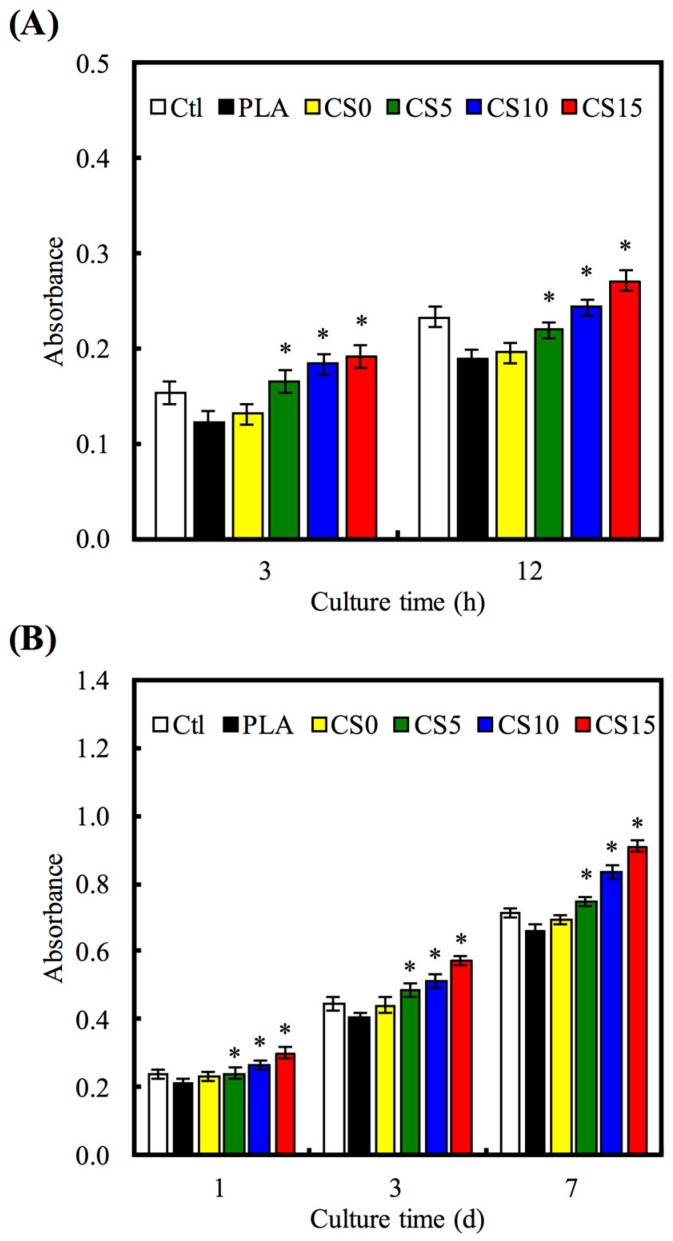
(**A**) Adhesion and (**B**) proliferation of hMSCs cultured on CS/CH-coated PLA mats for different time points. “*” indicates a significant difference (*p* < 0.05) compared to CS0.

The SEM micrographs of hMSCs adhesion on the CS/CH-coated PLA for 3 and 24 h are shown in Figure 8. Cells cultured on CS-coated specimens revealed higher area of cell adhesion compared to those on PLA with or without CH coating at each culture time period. The results of quantitative analysis of cell adhesion and proliferation are shown in Figure 9, illustrating that significantly higher amounts of cells adhered on CS-coated PLA than those on the pristine and CH-coated PLA at culture period of 3 and 12 h (Figure 9A). For example, CS5, CS10, and CS15 elicited significant increments of 25%, 40%, and 47%, respectively, in comparison with CS0 during the initial 3 h of culturing. Cell proliferation results showed significant differences (*p* < 0.05) in gradual increase of cell counts with increasing CS concentration when with the PLA and CH-PLA mats for all culture time periods (Figure 9B). 

**Figure 10:**
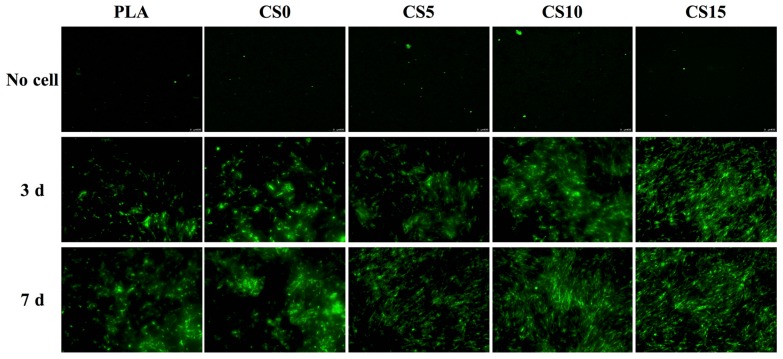
The immunofluorescence of hMSCs cultured on CS/CH-coated PLA mats for three and seven days.

The cell spreading and proliferation were also examined through immunofluorescent staining of F-actin. As shown in Figure 10, the fluorescence images demonstrated that majority of cells cultured on the PLA surface remained spherical and exhibited poor spreading, whereas a higher level of cell spreading into the typical polygonal morphology could be observed on CS15 after three days of culturing. It is worth noting that there is a confluence of a monolayer of cells on all CS-coated groups after seven days of culturing. In addition, the fluorescence was produced by from the cell itself, not from the auto-fluorescence of the scaffold or coating used. The presence of a suitable proteinaceous matrix as a receptor for cell adhesion such as integrins is critical for cell-biomaterial interactions. The formation of anchors between adhesion receptors and their ligands can trigger the formation of focal adhesion to further modulate the proliferation and differentiation processes through a series of signaling pathways activation [39]. Moreover, the released ionic products from CS could be responsible of the improvement of cell adhesion and proliferation. A previous study showed that the effective concentration range of Si ions capable of enhancing cell adhesion, proliferation, osteogenic differentiation, and mineralization is between 0.17 to 2.51 mM [40], which coincides with the results reported by Wu’s group [41]. In this regard, certain concentration of released Si ions from the CS/CH-PLA composite mats would enhance hMSCs proliferation.

### 3.5. Osteogenesis Gene Expression

**Figure 11:**
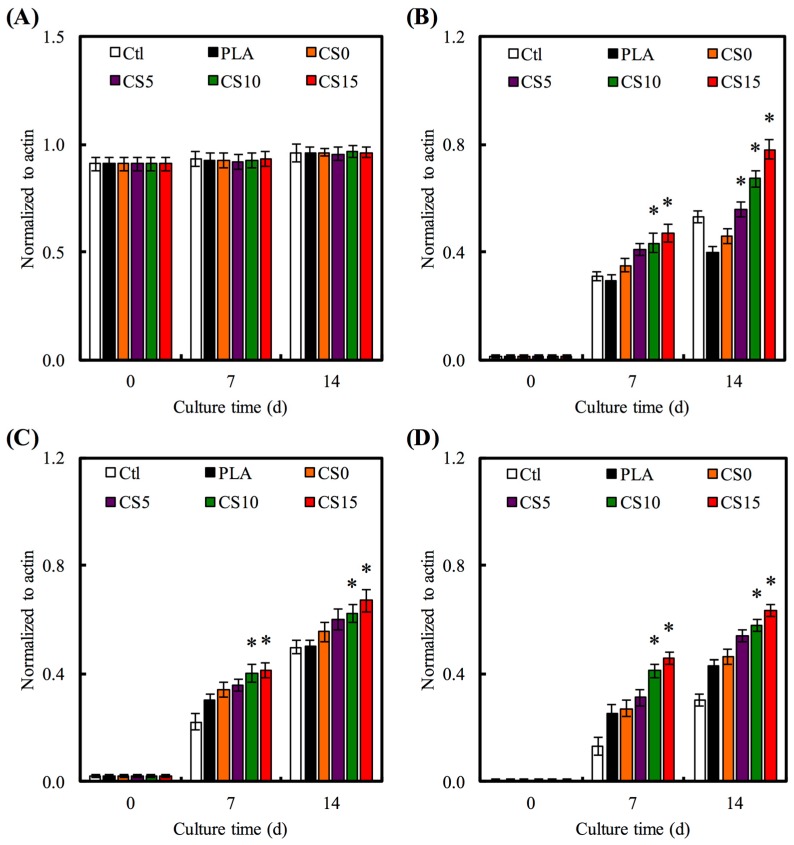
(**A**) COL; (**B**) alkaline phosphatase (ALP); (**C**) osteopontin (OPN); and (**D**) osteocalcin (OC) gene expression in the hMSCs were cultured on CS/CH-coated PLA mats for seven and 14 days. “*” indicates a significant difference (*p* < 0.05) compared to specimen without CS0.

The PLA mat is as a control to evaluate the effect of coated CS/CH on the expression of the osteogenic-related gene. There was no difference in COL I gene expression in all material groups after seven and 14 days (Figure 11A). According to the results shown in Figure 11, CS0 did not generate any stimuli on the specific ALP gene expression as compared to PLA. CS15 and CS20 generated higher specific ALP expression than PLA for all time points. Amongst other groups, CS15 showed the highest level for all genes, especially at day 14 where the level was as high as 1.7-, 1.3-, and 1.4-fold, for ALP, OPN, and OC, respectively, when compared to CS0. ALP is an early marker of hard tissue differentiation and it is generally accepted that an increase in osteoblast ALP equates to a more differentiated state [42]. OPN and OC are later makers of osteogenic differentiation. CS-contained specimens significantly promote osteogenesis-related gene expression (ALP, OPN, and OC) of hMSCs as compared to CH-coated PLA group. The positive effects of Si on the osteogenic differentiation of hMSCs and osteoblast cells have been proved by various studies [42,43]. In the present work, a sustained release of Si ions was observed from the CS/CH-coated PLA, which might account for the promoted osteogenesis differentiation of hMSCs cultured on the CS/CH-coated PLA mats. Wu et al. showed that CS-based biomaterials could promote osteogenesis differentiation and calcium deposition of human dental pulp cells and in vivo bone regeneration [44].

## 4. Conclusions

PLA mat was created via electrospinning, and CH/CS mixer was dropped on the mat surface as a simple method of surface coating. According to the SEM images shown, hMSCs had adhered homogenously onto the CS/CH-coated PLA mats, which indicated that CS15 displayed better apatite particulates deposition capability when immersed in DMEM. From the hMSCs study, it was confirmed that COL I and FN adsorption, cell adhesion, and proliferation were significantly increased on CS15 specimens. In vitro hMSCs cultivation on pure PLA and CS/CH-containing mats proved that the addition of inorganic component would not be toxic and promotes cell attachment ad proliferation, which leads to better osteogenesis. The results proved that this simple method of surface coating on bioactive and biodegradable nano-fiber using CS/CH is a feasible method in influencing cell behavior, which makes it a promising candidate for bone regeneration medicine.
